# 516. A Systematic Literature Review Evaluating Real-World Use of Nirmatrelvir-Ritonavir for the Prevention of COVID-19-Related Hospitalization and Death

**DOI:** 10.1093/ofid/ofad500.585

**Published:** 2023-11-27

**Authors:** Ashley S Cha-Silva, Meghan B Gavaghan, Jennifer L Nguyen, Ronika Alexander-Parrish, Jingyan Yang, Jaymin Patel, Denise A Garner, Richard Stanford, Genevieve Meier

**Affiliations:** Pfizer, Trumbull, Connecticut; Pfizer, Trumbull, Connecticut; Pfizer, Trumbull, Connecticut; Pfizer, Inc, Bowie, MD; Pfizer and Columbia University Institute for Social and Economic Research and Policy, New York, New York; AESARA, Chapel Hill, North Carolina; AESARA, Chapel Hill, North Carolina; AESARA, Chapel Hill, North Carolina; AESARA, Chapel Hill, North Carolina

## Abstract

**Background:**

Nirmatrelvir-ritonavir (NMV-r) is an oral antiviral medication used for the treatment of mild-to-moderate COVID-19 in patients aged 12 years or older at high risk of progression to severe disease and hospitalization. Following real-world utilization beginning in late 2021, millions of patients worldwide have been treated with NMV-r and many studies have described outcomes following treatment. This systematic literature review (SLR) was conducted to summarize the real-world clinical impact of NMV-r to inform healthcare decision-making.

**Methods:**

Real-world studies of NMV-r use available in English, with ≥5 subjects, and reporting on hospitalization and/or mortality outcomes were identified from Embase, PubMed, and relevant congress abstracts. Studies were evaluated for eligibility using population, intervention, comparison, outcome, study design criteria and time period (Dec 2021-Nov 2022) (Table 1). Dual-independent screening was used at the title, abstract and full-text levels with third reviewer consensus; data were extracted by a single reviewer with validation by a second reviewer prior to a quality rating assessment.

**Table 1.**
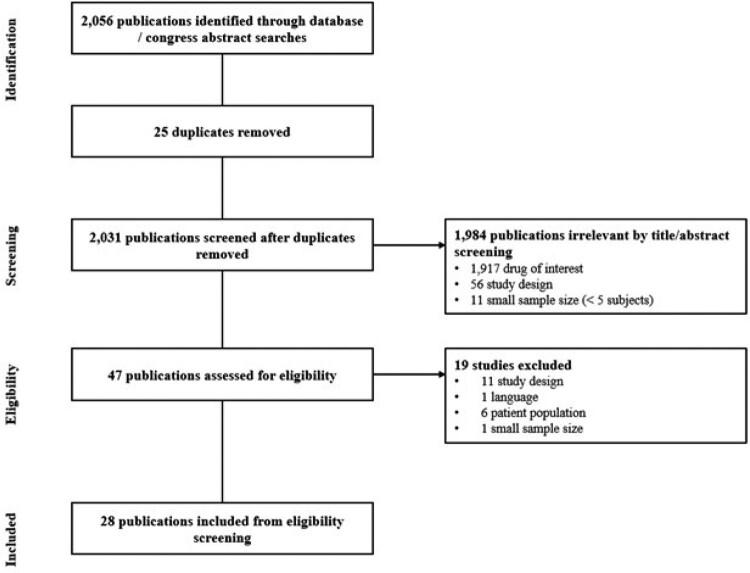
Population-Intervention-Comparators-Outcomes-Study Design Criteria

**Results:**

The SLR identified 28 eligible studies (Figure 1) representing 74,386 subjects that received NMV-r. The majority of studies reported on use in the United States (n=13) and patient vaccination status (n=25). Of the 16 studies that reported variant or sub-lineage data, all included patients treated during the Omicron period. Despite differences in baseline patient characteristics, treatment with NMV-r was consistently associated with a reduction in relative risk for hospitalization alone (n=5) or hospitalization and mortality (n=20). Studies that measured 30-day all-cause hospitalization (n=3) reported odds ratios (ORs) ranging from 0.43 to 0.54 (NMV-r vs. no NMV-r). Three studies measured 28-day all-cause mortality and reported ORs ranging from 0.05 to 0.23 (NMV-r vs. no NMV-r).

**Figure 1.**
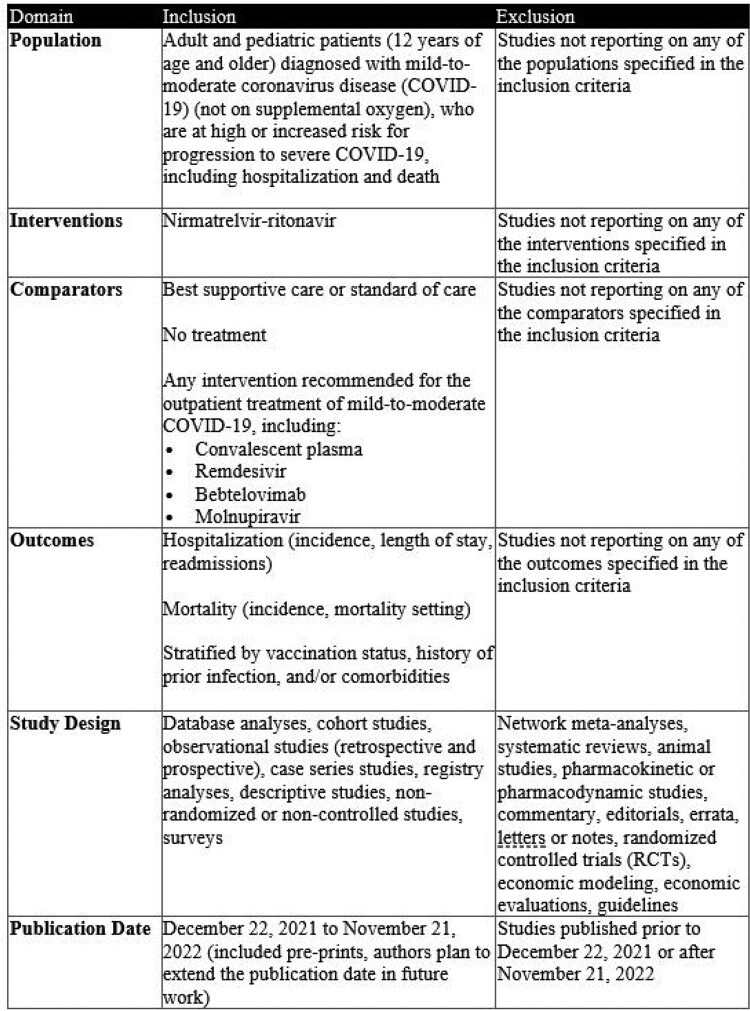
Preferred Reporting Items for Systematic Reviews and Meta-Analyses Flow Diagram

**Conclusion:**

Real-world studies show that NMV-r provides effective protection against hospitalization and death during the Omicron era among high-risk individuals. Outcomes following use of NMV-r in the real-world should be continually monitored as new studies are published and as the natural history of COVID-19 and treatment landscape continue to evolve.

**Disclosures:**

**Ashley S. Cha-Silva, PharmD, MS**, Pfizer Inc.: Employee|Pfizer Inc.: Stocks/Bonds **Meghan B. Gavaghan, MPH**, Pfizer: Advisor/Consultant **Jennifer L. Nguyen, ScD, MPH, MHCI**, pfizer: employee **Ronika Alexander-Parrish, RN, MAEd**, Pfizer, Inc.: Employee|Pfizer, Inc.: Stocks/Bonds **Jingyan Yang, MHS, DrPH**, Pfizer Inc.: Stocks/Bonds **Jaymin Patel, PharmD**, Pfizer: Advisor/Consultant **Denise A. Garner, PharmD**, Pfizer: Advisor/Consultant **Richard Stanford, PharmD, MS**, Pfizer: Advisor/Consultant **Genevieve Meier, PharmD, MSc, PhD**, Pfizer: Advisor/Consultant

